# Promising multifunctional van der waals heterostructure Ti_2_CO_2_/HfSi_2_N_4_ for photovoltaics and photocatalytic OER

**DOI:** 10.3389/fchem.2026.1849160

**Published:** 2026-06-24

**Authors:** Umair Mumtaz, Yi Ding, Ibrahim Khan, Shahid Iqbal, J. Andreas Larsson, Muhammad Sajjad

**Affiliations:** 1 Nottingham Ningbo China Beacons of Excellence Research and Innovation Institute, University of Nottingham Ningbo China, Ningbo, China; 2 Department of Chemical and Environmental Engineering, Faculty of Science and Engineering, University of Nottingham Ningbo China, Ningbo, China; 3 Applied Physics, Division of Materials Science, Department of Engineering Sciences and Mathematics, Luleå University of Technology, Luleå, Sweden; 4 Wallenberg Initiative Materials Science for Sustainability, Luleå University of Technology, Luleå, Sweden; 5 Laboratory of Carbonaceous Wastes Processing and Process Intensification Research of Zhejiang Province, University of Nottingham Ningbo China, Ningbo, China

**Keywords:** oxygen evolution reaction, photocatalysis, photovoltaics, thermoelectrics, Ti_2_CO_2_/HfSi_2_N_4_, type-I band alignment, vdW-heterostructure

## Abstract

This report presents an in-depth investigation of four stacking configurations of the van der Waals heterostructure (vdW-HS) of Ti_2_CO_2_ and HfSi_2_N_4_, conducted to explore their potential for green energy applications. The vdW-HS Ti_2_CO_2_/HfSi_2_N_4_ has a negligible lattice mismatch of 0.23% between the constituent monolayers, guaranteeing high structural compatibility. The dynamic stability has been confirmed by the phonon band structure, which has no imaginary frequencies throughout the full Brillouin zone. From the electronic band structure analyses, it has been confirmed that all stacking configurations yield identical band characteristics along with an indirect band gap of 0.88 eV calculated by using the Heyd−Scuseria−Ernzerhof (HSE06) functional with spin-orbit coupling (SOC). Remarkably, this vdW-HS displayed type-I band alignment, the electrons tunnel directly from the VBM to CBM of Ti_2_CO_2_ monolayer, allowing efficient carrier confinement and recombination, which is advantageous for advanced optoelectronic applications. Moreover, the electronic band edges of the vdW-HS Ti_2_CO_2_/HfSi_2_N_4_ demonstrate its high suitability for photocatalytic oxygen evolution reaction (OER), but not for hydrogen evolution reaction (HER). The latter unsuitability of the considered vdW-HS is also confirmed by ΔG_H_ > 0.2 or ΔG_H_ < −0.2 eV for all possible sites at the surface of the heterostructure. The considered vdW-HS has a significant static dielectric constant of 4.72, along with noticeable optical absorption in the visible spectrum and intense absorption of 1.50 × 10^6^ cm^-1^ in the ultraviolet region. The spectroscopic limited maximum efficiency (SLME) of ∼32% is higher than other highly appreciated thin-film photo-responsive absorber materials such as CuInSe_2_ (∼28%) and CdTe (∼31.5%). The n-type carriers have a higher value of Seebeck coefficient as compared to p-type carriers, which confirms that n-type doping will be more beneficial than p-type. The lattice thermal conductivity κ_ph_ of the vdW-HS Ti_2_CO_2_/HfSi_2_N_4_ is 8.41 W/mK at room temperature, which is at least 2.5 and 4.6 times lower than the lattice thermal conductivity of Ti_2_CO_2_ and HfSi_2_N_4_, respectively. These results highlight the potential of the vdW-HS Ti_2_CO_2_/HfSi_2_N_4_ as a highly suitable candidate for next-generation optical absorbers and thermoelectric materials for green energy technologies.

## Introduction

A broad range of two-dimensional (2D) materials has been successfully synthesized and widely studied for various applications since the discovery of graphene. The most studied 2D materials include silicon carbide, hexagonal boron nitride, MXenes, phosphorene, transition metal dichalcogenides (TMDC), and boron selenide. These 2D materials offer superior performance as compared to their bulk structure (Alam et al., 2021; Hou et al., 2016) due to their extraordinary physiochemical properties, such as high electrical conductivity, tunable band structures, and larger surface area, making them ideal for various applications to fulfil modern-day needs (Guo et al., 2023; Zhou et al., 2024a). The 2D materials like TMDC, black phosphorus, and MXenes possess notably higher carrier mobility and excitonic behavior. All these characteristics of 2D materials enable them to outperform in electronics, photonics, sensing, energy storage, coating material and biomedical technologies (Ghodrati et al., 2023; Li J. et al., 2024; Zhang et al., 2026).

The first MXenes Ti_3_C_2_T_x_ was produced by exfoliation of Ti_3_AlC_2_ in hydrofluoric acid at room temperature (Naguib et al., 2023). After this, the MXene family has expanded significantly, with more than 20 distinct structures successfully synthesized (Wang et al., 2024). The chemical formula for MXenes is M_n+1_X_n_T_x_, in which *M* is an early transition metal (e.g., Ti, Zr, Hf, V, Nb), X is C and N, *n* ranges from 1 to 4, and *T*_*x*_ denotes surface terminations such as O, OH, or F (Ahmadi et al., 2024; Shahzad et al., 2016). MXenes, comprising transition metal carbides, nitrides, or carbonitrides, are notable for their high electrical conductivity, mechanical robustness, large surface area, adjustable hydrophilicity, and optoelectronic versatility (Lin et al., 2017). MXenes also allow various surface functionalization, making them suitable for various catalytic activities (Wang X. et al., 2021). The compositional flexibility of MXenes leads to exceptional physical and chemical characteristics, making them suitable for various applications such as supercapacitors, electrodes for Li/Na-ion batteries, gas sensors, H_2_ storage, electro- and photo-catalysts, etc (Huang et al., 2018; Pang et al., 2019; Wang H. et al., 2018). Despite the traditional applications, recently MXene/cellulose aerogel has been used as support material for phase changing materials (Zhao et al., 2025). Among all MXenes, Ti_2_C possesses the lowest thickness, providing a larger surface area, which enhances its potential for surface-active processes (Guo et al., 2025). The physical features of Ti_2_C have been modified further by introducing various functional groups, such as Ti_2_CF_2_ and Ti_2_C(OH)_2_, which have metallic nature, although Ti_2_CO_2_ is a semiconductor in nature (Kurtoglu et al., 2012; Li et al., 2020). So, O-functionalized Ti_2_C has more possible applications as compared to Ti_2_CF_2_ and Ti_2_C(OH)_2_ (Come et al., 2012). Ti_2_CO_2_ has been successfully synthesized and characterized experimentally (Guo et al., 2025). Ti_2_CO_2_ has been reported as a good electrocatalyst for the carbon monoxide reduction (COER) process and handles the overall reaction highly efficiently (Guo et al., 2024; N et al., 2024). Due to the large surface area, Ti_2_CO_2_ allows various kinds of surface modifications, such as defect formation on the surface and functionalization, making it a good candidate for photocatalytic HER (Wang S. et al., 2018).

On the other side, HfSi_2_N_4_ belongs to the XZ_2_A_4_ family (Liu et al., 2021a), where X represents the transition metal, A is Group-IV element, and Z is a Group-V element, consisting of seven atomic layers with the sequence of A–Z–A–X–A–Z–A. In other words, an XA_2_ layer sandwiched between Z-A layers (Ahmad et al., 2025; Jappor et al., 2024). The HfSi_2_N_4_ monolayer is thermally, dynamically, and mechanically stable, and it is a direct band gap semiconductor with 2.71 eV band gap. The HfSi_2_N_4_ is a promising material for optoelectronics and nanoelectronics applications due to its direct band gap (Wang L. et al., 2021), excessive carrier mobility (Das et al., 2025), and high optical absorption in visible light. Furthermore, the bandgap can be accurately modulated by strain. By applying strain, it transforms from a direct to an indirect band gap semiconductor but also expands the absorption of visible light. HfSi_2_N_4_ is a good OER material (Das et al., 2025) and also a promising candidate for photocatalytic CO_2_ reduction (Yadav et al., 2021).

In the past few decades, several strategies have been successfully used to alter the physical characteristics of 2D materials, specially creating vacancies, doping, functionalization, applying external electric fields, strain engineering, and heterostructure formation, which can enhance their performance (Bafekry et al., 2024; Talib et al., 2025). Building vdW-HSs is also another practical option, which can effectively enhance the properties of 2D materials and offers a versatile platform for designing advanced materials through interfacial coupling (Ahmad et al., 2023; Alam et al., 2022; Din et al., 2018; Din et al., 2019; Din et al., 2021; Lan et al., 2022) The Transition metal dichalcogenides and transition metal oxides bested heterostructure have been studied abundantly, showing high catalytic activity (Tian et al., 2026). MXene-based 2D-vdW-HSs such as Ti_2_CO_2_/Zr_2_CO_2_, Ti_2_CO_2_/MoS_2_, exhibit extraordinary electrochemical features (Nasrin et al., 2022). The study of WSe_2_/Ti_3_C_2_T_x_ MXene based heterostructure opens new avenue to the exploration of functionalization and the stacking order (Hou et al., 2026). Sc and Y-doped Ti_2_CO_2_/MoS_2_ have been reported as potential anode materials with high energy density, which are highly suitable for efficient quantum capacitance (Zhou et al., 2024b). The type-II band alignment g-C_3_N_4_/Ti_2_CO_2_ heterostructure has significant charge transfer from g-C_3_N_4_ to Ti_2_CO_2_ making it suitable for photocatalytic applications (Liu et al., 2021b). The MoTe_2_/Ti_2_CO_2_ z-type heterostructure has high light absorption properties and overall photocatalytic water splitting (Nirjhar et al., 2023). Similarly, MA_2_Z_4_-based vdW-HS has a huge potential for applications because of its high tunability in structural composition and physical properties (Latychevskaia et al., 2024). MoSe_2_/MoSi_2_P_4_ and PtS_2_/MoSi_2_P_4_ vdW-HS have shown remarkable performance as photovoltaics and photocatalytic HER (Saira N. et al., 2025; Saira I. A. et al., 2025). On the other hand, the self MA_2_Z_4_ based vdW-HS has high performance and wide bandgaps (Bao et al., 2023; Li et al., 2026). In recent years, different 2D materials have been incorporated with each other to design highly efficient heterostructures. However, designing MXenes/MA_2_Z_4_ heterostructures is one of the unexplored combinations that can outperform in modern electronic devices. The MXene/MoSi_2_N_4_ (M_n+1_X_n_T_2_, M = Ti–Ta; n = 1 and 2; X = C and N; T = F, O, and OH) heterostructure has strong Fermi level pinning effect which results in low contact resistance. This repost is one of the few report that outlines the potential of MXene and MA_2_Z_4_-based vdW-HSs (Wang et al., 2023). So far no, reports discuss the detailed study of optoelectronic and photocatalytic response of MXene/MA_2_Z_4_ vdW-HS.

Inspired by the unique and tunable properties inherent to vdW-HSs and the huge research gap in the exploration of MXenes/MA_2_Z_4_, in this work, we have constructed a vdW-HS of 2D materials, Ti_2_CO_2_ and HfSi_2_N_4_, from two different families having type-I band alignment which can perform well for photocatalytic application. So, we employed DFT to investigate the structural, vibrational, thermodynamic, mechanical, charge density difference, electronic, photocatalytic, optical, and thermoelectric properties of the novel vdW-HS Ti_2_CO_2_/HfSi_2_N_4_ and disclosed its potential for green energy and photocatalysis applications.

## Computational details

The *ab initio* calculations are performed by using the VASP code (Hafner, 2008; Kresse and Furthmüller, 1996), utilizing the projector augmented wave (PAW) along with the Perdew−Burke−Ernzerhof (PBE) pseudopotentials (Perdew et al., 1996). The exchange-correlation functional is discussed with the PBE generalized gradient approximation and the HSE06 functional with SOC and without-SOC effect to attain the precise band gap. A cutoff energy of 550 eV and Monkhorst–Pack *k*-mesh of 9 × 9 × 1 (15 × 15 × 1) are used for the self-consistent (non-self-consistent) calculations. The DFT-D3 method of Grimme (Grimme et al., 2010; Grimme et al., 2011) is used to correct van der Waals interactions in vdW-HS Ti_2_CO_2_/HfSi_2_N_4_. The use of DFT-D3 for the simulations of the individual monolayers (Ti_2_CO_2_ and HfSi_2_N_4_) in the literature has been reported numerous times. To ensure a consistent and meaningful comparison when forming the heterostructure, it is necessary to use the same theoretical framework. Using a different functional or vdW correction for the heterostructure would introduce a methodological inconsistency, making the comparison invalid. Our calculations for the isolated monolayers using the DFT-D3 method successfully reproduce the key structural and electronic properties (e.g., lattice constants, band gaps) reported in the literature (Ali et al., 2023; Hua et al., 2024; Yu et al., 2015). This confirms that the parameterization is well-suited for the constituent materials of our heterostructure, giving us high confidence in its application to the heterostructure. Structural relaxation is carried out until the Hellmann−Feynman forces on all atoms drop below 3 meV/Å and the total energy converged between two consecutive self-consistent electronic cycles was less than 1 × 10^−6^ eV. An out-of-plane vacuum of 15 Å is added to prevent artifacts due to the periodic boundary conditions. The phonon dispersions were obtained for a 4 × 4 × 1 supercell in the Phonopy code (Togo and Tanaka, 2015) by utilizing 3 × 3 × 1 *k-*mesh. Furthermore, the *ab initio* molecular dynamics (*ai*MD) simulations were performed at 300 K and 500 K temperatures using the 4 × 4 × 1 supercell of the vdW-HS Ti_2_CO_2_/HfSi_2_N_4_ to confirm the thermal stability. For *ai*MD simulations, we used the Nose-Hoover thermostat using the NVT ensemble for 14 ps, with a time step of 2 fs. The thermoelectric properties have been calculated by using the BoltzTraP2 code (Mad et al., 2018) with a 60 × 60 × 1 dense K-mesh. VASPKIT has been used for pre- and post-processing purposes, especially for SLME calculation from absorption coefficient and band gaps (Geng et al., 2025; Wang V. et al., 2021).

## Result and discussion

**FIGURE 1:**
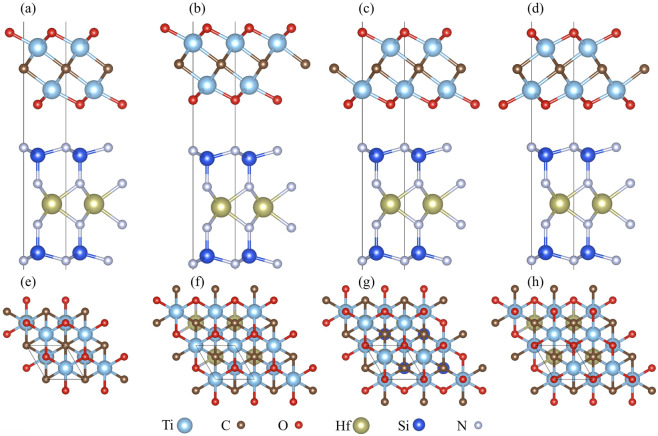
Side (1^st^ row) and top (2^nd^ row) views of **(a,e)** AA-stacking, **(b,f)** AB-stacking, **(c,g)** AC-stacking, and **(d,h)** AD-stacking of the vdW-HS Ti_2_CO_2_/HfSi_2_N_4_. The black rectangles enclose the unit cells, respectively.

The optimized structures of vdW-HS Ti_2_CO_2_/HfSi_2_N_4_ with AA-, AB-, AC-, and AD-stacking configurations are shown in Figures 1a–h. The heterostructure in all the stackings maintains the hexagonal phase geometry, consistent with the symmetry of the constituent monolayers Ti_2_CO_2_ and HfSi_2_N_4_. The optimized lattice parameters (*a*) for all four stackings and constituent monolayers are listed in Table 1, which are consistent with previously reported values and suggests that our approach for structural optimization is reliable. A slight lattice mismatch (∼0.23%) between the constituent monolayers would facilitate the epitaxial growth of vdW-HS Ti_2_CO_2_/HfSi_2_N_4_ (Singh N. B. et al., 2024). As far the possible synthesis route is concerned, Ti_2_CO_2_ can be synthesized by the exfoliation of Ti_2_C MAX phases, furthermore, HfSi_2_N_4_ can also be synthesized using Chemical vapor deposition as discussed by Yi-Lun Hong and his colleagues. After synthesizing individual monolayer, the vdW heterostructure can be synthesized by epitaxial growth, exfoliation, and physical/chemical vapor deposition PVD/CVD (Hong et al., 2020; Lu et al., 2023; Yang et al., 2020).

**TABLE 1:** The optimized lattice parameters (a), interlayer distances (d), binding energy (E_b_), formation energy (E_form_), band gap (E_g_) for the vdW-HS Ti_2_CO_2_/HfSi_2_N_4_ and its constituent monolayers.

*Stacking*	*a (Å)*	*d (Å)*	*E*_*b*_ *(eV)*	*E*_*f*_ *(eV)*	*E*_*g*_ *(eV) PBE-SOC*	*HSE-SOC*	*Literature*	
							*a (Å)*	*E*_*g*_ *(eV)*
*Ti*_*2*_ *CO*_*2*_	3.019	-	-	-	-	0.88	3.03	0.89 (Hua et al., 2024)
							3.02	0.86 (Ali et al., 2023)
							3.03	0.88 (Yu et al., 2015)
*HfSi*_*2*_ *N*_*4*_	3.012	-	-	-	-	2.71	3.01	2.89 (Das et al., 2025)
							3.02	2.81 (Yang et al., 2024)
							3.02	2.90 (Yang et al., 2021)
*AA*	3.015	2.66	−0.271	−0.270	0.226	-	-	-
*AB*	3.013	3.13	−0.190	−0.189	0.224	-	-	-
*AC*	3.015	2.68	−0.263	−0.262	0.225	-	-	-
*AD*	3.016	2.62	−0.284	−0.283	0.221	0.89	-	-

The ground-state energy analysis reveals an energy difference of less than 30 meV between AA, AC, and AD-stacking configurations, with the AD-stacking configuration being the most energetically favorable, exhibiting the lowest ground-state energy. On the other hand, the AB configuration is the least favorable, having the highest ground state energy as compared to the other three configurations. The binding energy also gives information about the structural stability of the studied vdW-HS. The binding energies of AA-, AB-, AC-, and AD-stacking are −0.27, −0.19, −0.26, and −0.28 eV, respectively. The calculated formation energy (E_f_) also follows a similar trend to binding energy. Here, we also see that the binding energy of AD-stacking is minimum, which is incorporated by the smallest interlayer distance of 2.61 Å. Similarly, the binding energy of AB-stacking is maximum, having the highest interlayer distance of 3.13 Å among all other stacking. This difference in the energies and interlayer distances is due to the difference in the strength of interatomic interactions of atoms of each layer. So, the AD-stacking is energetically more stable than all other (Opoku et al., 2022). The interlayer interaction in vdW-HS Ti_2_CO_2_/HfSi_2_N_4_ is dominated by vdW forces, as evidenced by the equilibrium distances (∼2.62–3.13 Å) and the binding energies being comparable to typical vdW-HS heterostructures (Novoselov et al., 2016; Shi et al., 2024).

**FIGURE 2:**
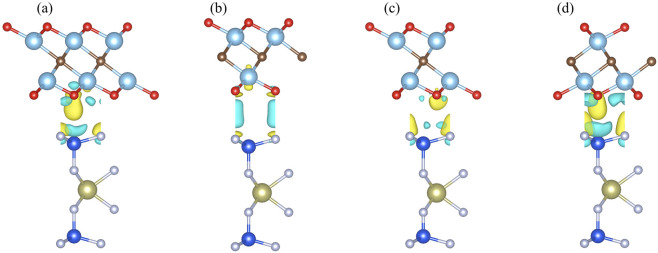
The isosurface charge density difference of the **(a)** AA-stacking, **(b)** AB-stacking, **(c)** AC-stacking, and **(d)** AD-stacking configuration of the heterostructure Ti_2_CO_2_/HfSi_2_N_4_. Cyan and yellow regions signify charge depletion and accumulation, respectively.

We investigate the interlayer electronic interactions within the Ti_2_CO_2_/HfSi_2_N_4_ heterostructure by calculating charge density difference isosurfaces across AA-, AB-, AC-, and AD-stacking configurations (Chen et al., 2023; Li L. et al., 2024; Li et al., 2025). In Figures 2a–d, the charge density difference isosurfaces reveal charge redistribution between the Ti_2_CO_2_ and HfSi_2_N_4_ monolayers. The significant charge redistribution occurred at the interface between Ti_2_CO_2_ and HfSi_2_N_4_; the cumulative charge accumulation of 0.01–0.02 electrons has been calculated from the Bader charge analysis on Ti_2_CO_2_, depleted from HfSi_2_N_4_, which verifies that the type of interaction is a van der Waals interaction. This charge redistribution introduced the interfacial dipole at the interface, which is mainly responsible for the modulation of optoelectronic characteristics of the heterostructure as compared to the constituent monolayers. This type of charge redistribution or electron-hole recombination at the interface usually occurs in the vdW-HS with type-I band alignment, which has been confirmed in the band alignment section. The negligible of interlayer electron transfer of (0.01–0.02 e) from Ti_2_CO_2_ and HfSi_2_N_4_ demonstrates the vdW forces in the heterostructure and negligible hole transfer from Ti_2_CO_2_ makes HfSi_2_N_4_ more suitable for the OER which requires holes. The transfer of holes before the recombination which usually occurs in type-I band alignment heterostructure also predicts the high charge mobility, transferring holes to the interface before the recombines.

**FIGURE 3:**
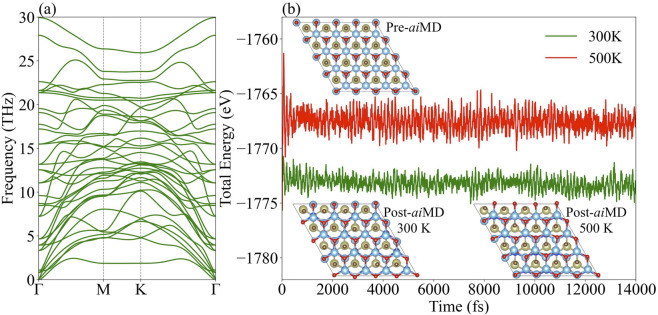
**(a)** Calculated phonon band structures for AD-stackings of the vdW-HS Ti_2_CO_2_/HfSi_2_N_4_ and **(b)** total energy during aiMD simulations of the AD-stacking configuration of the vdW-HS Ti_2_CO_2_/HfSi_2_N_4_ at 300K and 500K, including the pre (at top) and post-aiMD simulation structure (at bottom).

The phonon band structure of AD-stackings of vdW-HS Ti_2_CO_2_/HfSi_2_N_4_ is displayed in Figure 3a. Notably, no imaginary frequency appears throughout the Brillouin zone, which confirms that AD-stacking of vdW-HS Ti_2_CO_2_/HfSi_2_N_4_ is dynamically stable. 1t is worth noticing that there is a significant overlap between acoustic (the first three bands) and low-lying optical phonon modes, suggesting that this heterostructure is likely to have a low lattice thermal conductivity (Sajjad and Singh, 2022) and thus holds potential applications in thermoelectrics.

As shown in Figure 3b, the energy trajectory obtained from *ai*MD simulations of the AD-stacked configuration validates robust thermal stability at 300 K and 500 K. Following an initial equilibration period, the total energy converges to a stable equilibrium with slight fluctuations, with a stable structure after a time duration of 14 ps? Comparative structural analysis between the initial and final structure reveals no bond breakage and negligible distortion between the pre- and post-*ai*MD simulation structures, validating thermal resilience of the heterostructure.

The mechanical stability of all possible stacking configurations of the considered vdW-HS is thoroughly evaluated by evaluating primary elastic parameters, including the elastic stiffness constants, Poisson’s ratio, Bulk, Shear, and Young’s modulus. The computed elastic stiffness constants C_ij_ satisfy the Born-Huang stability criteria (Singh S. et al., 2021), confirming the mechanical stability of the vdW-HS Ti_2_CO_2_/HfSi_2_N_4_, as listed in Table 2. The high values of the Bulk, Shear, and Young’s modulus are associated with the high resistance to elastic deformation and reflect strong interatomic bonding. Additionally, the Poisson’s ratio for all configurations is less than 0.5, which is consistent with the preceding studies on stable 2D materials (Huang et al., 2020; Khandy et al., 2018; Lee et al., 2018), validating the structural robustness of the considered vdW-HS. The stability of vdW-HS Ti_2_CO_2_/HfSi_2_N_4_ across all possible stacking configurations is worth noticing because it enhances its versatility and practicality for advanced technological applications (Geim and V Grigorieva, 2013). These mechanical characteristics of the considered vdW-HS provide greater flexibility during synthesis, allowing for tunable properties, and confirm resilience under working conditions, making these a highly promising candidate for a large variety of applications.

**TABLE 2:** The calculated elastic constants (C_ij_), bulk modulus (B), shear modulus (S), Young’s modulus (Y), and Poisson’s ratio (P) of all four stacking configurations of the vdW-HS Ti_2_CO_2_/HfSi_2_N_4_.

*Configurations*	*C*_*11*_	*C*_*12*_	*C*_*66*_	*B (N/m)*	*S (N/m)*	*Y (N/m)*	*P*
*AA*	728.70	728.70	251.73	476.96	251.73	659.08	0.3
*AB*	735.31	735.31	254.57	480.73	254.57	665.75	0.3
*AC*	721.27	721.27	250.40	470.87	250.40	653.88	0.3
*AD*	721.32	721.32	250.55	470.76	250.55	654.09	0.3

**FIGURE 4:**
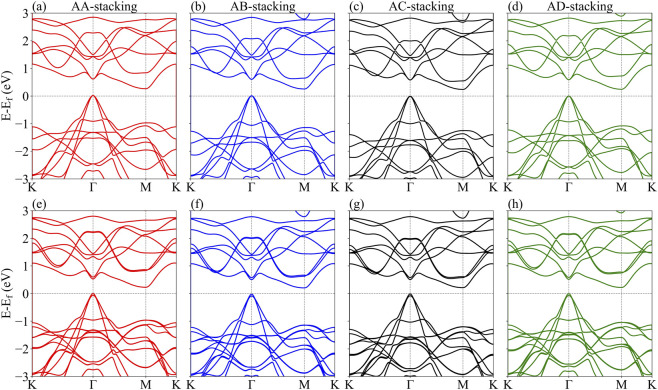
Calculated electronic band structures for **(a)** AA-, **(b)** AB-, **(c)** AC- and **(d)** AD-stacking of vdW-HS without-SOC (1^st^ row) along with electronic band structures for **(e)** AA-, **(f)** AB-, **(g)** AC- and **(h)** AD-stacking of vdW-HS Ti_2_CO/HfSi_2_N_4_ with-SOC (2^nd^ row) at the PBE level. The Fermi level is set at 0 eV.

**FIGURE 5:**
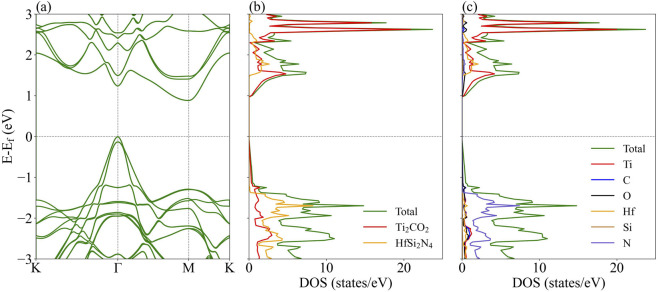
**(a)** Calculated electronic band structure, **(b)** TDOS, and **(c)** Elemental-PDOS of AD-stacking vdW-HS Ti_2_CO_2_/HfSi_2_N_4_ at HSE06-SOC level.

Figure 4 displays the electronic band structure of all four stackings of vdW-HS Ti_2_CO_2_/HfSi_2_N_4_, revealing identical features, including an indirect band gap of 0.22 eV (0.25 eV) at the PBE level with (without) SOC inclusion, accompanied by similar band dispersions. SOC introduces minimal band splitting of 0.08 eV in the first two valence bands. Notably, the presence of an indirect band gap renders this material promising for optoelectronic applications (Malyi and Acosta, 2020). Since the PBE functional underestimates the band gap value (Meunier and Robertson, 2021), we engaged the hybrid functional HSE06 for accurate band gap determination through band gap and density of states (DOS). The obtained findings are given in Figure 5 for AD-stacking. The calculated band gap of vdW-HS Ti_2_CO_2_/HfSi_2_N_4_ appears to be 0.89 eV with HSE06+SOC. Here, the band splitting also occurs with the same trend. The DOS analysis reveals that the conduction band minimum (CBM) and the valence band maximum (VBM) are composed of Ti *d*-orbital sand O *p*-orbitals of Ti_2_CO_2_, predicting the type-I band alignment. Additionally, Hf *d*-states contributed significantly to the formation of the conduction band and N *p*-orbitals of HfSi_2_N_4_ to the formation of valance band, but relatively far from the Fermi level. The strong hybridization between Si *p*-, O *p*-, and C *p*-states has been witnessed across both the valence and conduction bands. The low effective mass of electrons and holes derived from the curvature of the band structure are 0.66 and 0.55, these relatively light as compared to HfSi_2_N_4_ (1.16 and 2.02 (Das et al., 2025)) and Ti_2_CO_2_ (0.87 and 1.32 (Munawar et al., 2021)). The nearly balanced and comparatively low effective masses imply high carrier mobilities and long diffusion lengths, which is advantageous for efficient charge extraction in photovoltaic, photocatalytic, and photodetector devices. The absence of significant effective-mass anisotropy near the band edges further indicates isotropic charge transport, which facilitates uniform carrier collection across the interface (Czyz and Snee, 2025). The light hole mass ensures high hole mobility as compared to the electrons, reduces the transit time to the surface and minimizing recombination, which is advantageous for the vdW-HS, where holes are not spatially separated from electrons by a built-in field; instead, they diffuse to the surface and react before recombining. The small effective mass thus directly supports the high photogenerated hole potential of in driving the OER, as the rapid hole transport ensures that the energetic driving force is not wasted on slow carrier dynamics.

**FIGURE 6:**
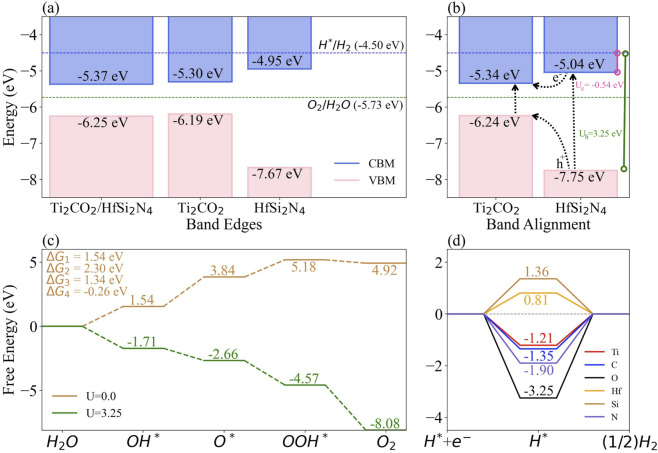
Calculated **(a)** band edge positions relative to the vacuum level along with **(b)** the band alignment, **(c)** Gibbs free energy OER (ΔG_OER_), and **(d)** Gibbs free energy HER (ΔG_H_) for AD-stacking of vdW-HS Ti_2_CO_2_/HfSi_2_N_4_.

Photocatalytic materials appropriate for hydrogen evolution must fulfill key energetic criteria: the CBM must be higher than the hydrogen reduction potential (H^+^/H_2_), and the VBM must be below the oxygen oxidation potential (O_2_/H_2_O), ensuring thermodynamic feasibility for overall water splitting (Xiong and Yang, 2023). This configuration enables photogenerated electrons and holes to effectively participate in the hydrogen and oxygen evolution reactions, respectively. When compared to the standard redox potentials of water (see Figure 6a), the CBM lies below the H^+^/H_2_ reduction level (−4.50 eV), indicating insufficient driving force for hydrogen evolution, while the VBM is significantly below the O_2_/H_2_O oxidation potential (−5.73 eV), suggesting that the material is capable of oxidizing water to produce oxygen. Despite this favorable alignment for the oxygen evolution reaction, the suboptimal CBM renders the material ineffective for overall water splitting. Additionally, the narrow band gap restricts light absorption primarily to the infrared region, which is not ideal for solar energy harvesting. To overcome these limitations and enhance photocatalytic performance, several strategies may be considered, including band edge engineering through anion or cation substitution, surface functionalization, or application of tensile strain to upshift the CBM. The prediction of vdW-HS Ti_2_CO_2_/HfSi_2_N_4_ as a photocatalyst for OER is mainly based on its band edges and band alignment, leading to outstanding carrier confinement properties required for photocatalytic OER, which are critical initial filters for classifying potential photocatalysts before embarking on resource-intensive catalytic mechanistic studies. This prediction encouraged us to study the reaction mechanism, which has been discussed below.

Figure 6b confirms that vdW-HS Ti_2_CO_2_/HfSi_2_N_4_ displays type-I (straddling gap) band alignment, such that Ti_2_CO_2_ provides CBM and VBM positioned between the CBM and VBM of HfSi_2_N_4_. Thus, the type-I band alignment is observed in the vdW-HS Ti_2_CO_2_/HfSi_2_N_4_, which enables efficient carrier confinement and recombination, making it suitable for optoelectronic applications. In such a vdW-HS, the relaxation of photoexcited electrons and holes is incorporated in the same material, specifically, in this case, Ti_2_CO_2_ is that material. This process of electron-hole pair recombination results in the formation of interlayer excitons, which trap free carriers at the interface, suppressing the interlayer photocurrent. The type-I band alignment can repel carriers inside the materials, which is helpful to avoid carrier loss from the surface of the heterostructure. This carrier confinement in type-I band alignment improves the carrier lifetime, which significantly enhances the efficiency of the optoelectronic device (Luo et al., 2021). The resulting enhancement due to type-I band alignment emphasizes the potential for an efficient heterostructure for potential optoelectronic applications. Furthermore, the low effective mass, high mobility of holes and negligible hole transport on HfSi_2_N_4_ due to the interfacial dipole makes this type-I band alignment heterostructure a good photocatalyst. Due to long carrier lifetime and high mobility, the holes can easily transport from Ti_2_CO_2_ to the HfSi_2_N_4_, which is further supported by the strong potential of photogenerated holes making it favorable for photocatalytic OER. The similar characteristics has already been observed in other type-I band alignment photocatalysts such as Fe_2_O_3_-TiO_2_ (Singh A. P. et al., 2021), CdS-In(OH)_3_ (Chava et al., 2022) and BX-ZnO (X = P, As) (Do et al., 2020).

The photocatalytic OER activity of vdW-HS Ti_2_CO_2_/HfSi_2_N_4_ is investigated in terms of its Gibbs free energy, as shown in Figure 6c. OER contains intermediate states of ^*^OH, ^*^O, and ^*^OOH absorbed on the surface. The ground state energies of the water adsorbed heterostructure on the Ti_2_CO_2_ and HfSi_2_N_4_ sides are almost the same. So, the feasibility of the catalytic pathway of the OER reaction has been explored on both surfaces of vdW-HS Ti_2_CO_2_/HfSi_2_N_4_ at each distinct site. The Si-site is the active site during the OER process, having the least overpotential as compared to the other sites. During the OER, the Si-atom slightly moves outward from the HfSi_2_N_4_ after structural optimization for each reaction intermediate, which recovers after the production of the O_2_ molecule on the surface. Among the four steps of OER, the reaction for ^*^OH into ^*^O is the rate-determining step with ΔG of 2.30 eV which means the calculated overpotential is 1.07 V at U = 0 V vs. the normal hydrogen electrode NHE. This overpotential is significantly high as compared to expensive metal oxides IrO_2_ and RuO_2_, while this overpotential is competitive for other emerging transition metals-based materials such as MoS_2_ (1.62 V) (German and Gebauer, 2023) and CoSAs-MoS_2_ (Luu Luyen Doan et al., 2021). However, the strong oxidation potential provided by photo-generated holes (U = 3.25 V) can provide the necessary deriving force to overcome the overpotential, making the whole OER occur spontaneously, which confirms that vdW-HS Ti_2_CO_2_/HfSi_2_N_4_ is feasible for photocatalytic OER.

A value of ΔG_H_ close to zero is ideal for the hydrogen evolution reaction (HER). In other words, the 2D materials having |ΔG_H_| between −0.2 and 0.2 eV are generally referred to as high-class catalysts for HER (Sajjad et al., 2023). We absorbed H on each distinct possible site of absorption on the considered vdW-HS. Figure 6d also illustrates that vdW-HS Ti_2_CO_2_/HfSi_2_N_4_ does not perform very well as a HER material, which confirms the prediction made from the band edges positioning relative to the vacuum level. The simulated values of ΔG_H_ for the considered vdW-HS are significantly displaced from the thermoneutral point (ΔG_H_ ≈ 0 eV), which is considered not to be optimal for efficient HER catalysis. Specifically, high positive ΔG_H_ values indicate the weak binding of hydrogen at the surface of the catalyst, leading to insufficient proton adsorption required for HER. Contrarily, highly negative values of ΔG_H_ signify the strong binding of hydrogen at the surface, which hinders the subsequent desorption of molecular hydrogen (H_2_) and limits the overall catalytic activity. All these values of ΔG_H_ for HER suggest that vdW-HS Ti_2_CO_2_/HfSi_2_N_4_ lacks the necessary thermodynamic conditions to facilitate the HER efficiently. As such, the material may not serve as an effective tool for HER without further modification or optimization (Kumatani et al., 2019).

**FIGURE 7:**
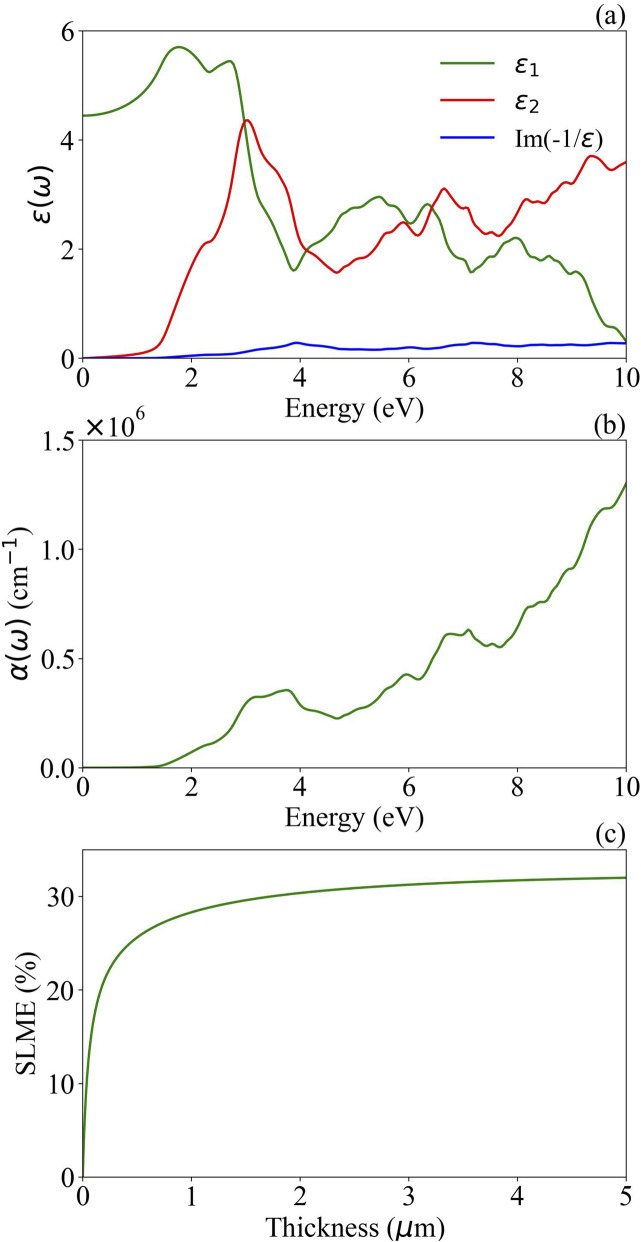
Calculated **(a)** dielectric function ε, **(b)** absorption coefficient α, and **(c)** SLME as a function of the film thickness of AD-stacking of the vdW-HS Ti_2_CO_2_/HfSi_2_N_4_ at HSE06-SOC level.

Next, we explored the optical properties of vdW-HS Ti_2_CO_2_/HfSi_2_N_4_. Figure 7a displays the real (ε_1_) and imaginary (ε_2_) parts of the dielectric function. The static dielectric constant (i.e., ε_1_ at zero frequency) is found to be 4.76, indicating a significantly moderate electronic polarizability of the heterostructure. A pronounced peak resides at 1.02 eV, corresponding to the highest polarization within the visible region (Khalid et al., 2024; Singh D. et al., 2024). The imaginary part ε_2_ reveals the absorptive nature of the heterostructure, which is related to the interband transition occurring in the material. The ε_2_ starts to rise after 1.02 eV, where the transitions start to occur after the absorption of high-energy photons exceeding the threshold limit of the optical bandgap from O-*p* to Ti-*d* state, as identified by PDOS, where these states mainly dominate the VBM and CBM. The highest ε_2_ peak resides at 2.9 eV, after which it decreases, and so does the rate of interband transitions. The dielectric loss factor Im (-1/ε) (Gong et al., 2023; Shirley, 2020) has also been displayed in Figure 7a, which shows the energy dissipation in vdW-HS Ti_2_CO_2_/HfSi_2_N_4_. The maximum dielectric loss factor of 0.28 has been grasped, showing low energy dissipation leading to better performance.

Figure 7b shows the absorption spectrum of vdW-HS Ti_2_CO_2_/HfSi_2_N_4_, revealing that significant adsorption starts in the visible region at 1.7 eV. However, the highest overall absorption of 1.50 × 10^6^ occurs in the UV region. Hence, the absorption of visible and UV light is mainly responsible for the electron-hole pairs. One can observe that absorption begins at around 1.02 eV, which is close to the heterostructure band gap, enabling the generation of electron-hole pairs and, hence, activating the photovoltaic effect. With a broad absorption range, the Ti_2_CO_2_/HfSi_2_N_4_ vdW-HS is highly suitable for photovoltaic applications.

In Figure 7c, the SLME of vdW-HS Ti_2_CO_2_/HfSi_2_N_4_ has been shown as a function of layer thickness, which is one of the main parameters of huge importance for estimating solar cell efficiency. The SLME is mainly affected by the material’s electronic band gap and its optical absorption (Singh and Ahuja, 2022). As displayed in Figure 7c, the SLME increases linearly with the increasing material’s thickness. Despite the inherent limitations of low indirect bandgap (0.89 eV), the phonon-assisted transitions and high exciton binding energies significantly enhance their light absorption and emission efficiencies, achieving high SLME values comparable to direct-bandgap materials. The SLME is not solely dependent on the strength of absorption at the band edge but is a complex function of the detailed absorption profile, non-radiative recombination losses, and the material’s ability to extract high potential of (3.25 V) photogenerated carriers, which was formulated by Yu and Zunger (2012). The indirect gap of 0.89 eV is not only considered for SLME calculation, but also the direct allowed band gap, which is 1.45 eV. The combined effect of the indirect band gap, direct allowed band gap, and optical band gap makes it near-optimal for a single junction. Many direct-gap 2D materials suffer from strong excitonic effects that can reduce the usable photovoltage. The type-I band alignment, promoting carrier confinement and reducing interface recombination support efficient charge collection, and the exciton binding energy calculated from E_exciton_ = E_opt_-E_g_ is 0.14 eV, demonstrating that the low exciton binding energy facilitates the efficient extraction of photogenerated carriers before they can recombine in the type-I band alignment, minimizing losses and boosting the fill factor and open-circuit voltage in the SLME simulation. The vdW-HS Ti_2_CO_2_/HfSi_2_N_4_ reaches a peak efficiency of 32% at a film thickness of 5 μm. This maximum SLME value is compared with the other highly appreciated photovoltaic materials, like silicon (Oh et al., 2015)^,^ (Lee et al., 2014), and other extensively used thin-film solar cell absorbers such as CuInSe_2_ (∼28%) and CdTe (∼31.5%) (Sharan et al., 2022). All these results don’t predict the device performance, however, the high absorption coefficient and SLME positions vdW-HS Ti_2_CO_2_/HfSi_2_N_4_ as highly efficient photo-absorber to be used in next-generation optoelectronic devices which should be explored experimentally.

**FIGURE 8:**
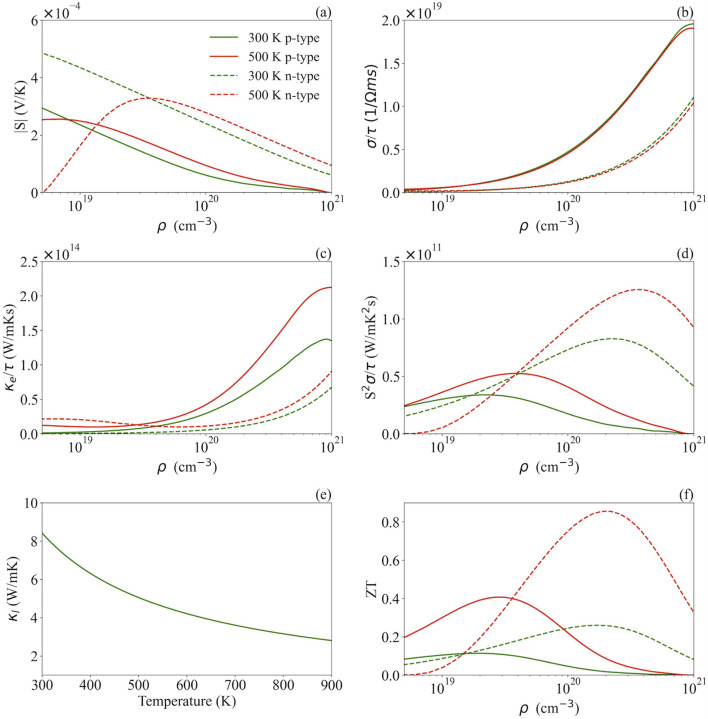
Calculated **(a)** the Seebeck coefficient S, **(b)** electrical conductivity σ/τ, **(c)** the electronic thermal transport coefficients κ_e_/τ, **(d)** power factor, and **(f)** figure of merits as a function of carrier concentration (ρ) at 300 and 500 K. **(e)** Lattice thermal conductivity as a function of temperature.

We have also calculated the thermoelectric features of AD-stacking of Ti_2_CO_2_/HfSi_2_N_4_ as a function of their carrier concentration (ρ), plotted in Figure 8. In Figure 8a, the Seebeck coefficient S is plotted at 300 K and 500 K temperatures with respect to varying carrier concentrations. At 300 K, S decreases nearly linearly with increasing carrier density, revealing typical semiconductor behavior. In contrast, S initially rises with increasing carrier concentration due to thermally activated transport at 500 K temperature and a broader energy distribution of carriers. This trend converses at higher carrier concentrations due to degeneracy effects suppressing the Seebeck effect. In both cases of 300K and 500 K for n-type carriers, S values are consistently higher than the S values of p-type carriers. The significantly higher values of S for n-type carriers confirm the higher effective masses of electrons as compared to holes, leading to the fact that n-type doping will lead to a higher Seebeck coefficient as compared to p-type (Gandi et al., 2016). S has an inverse relation with the electrical conductivity (σ/τ) and the thermal conductivity (κ_e_/τ), which can be clearly observed in Figures 8b,c.

Both n-type and p-type charge carriers can contribute to the electrical σ/τ and thermal conductivity κ_e_/τ, so both σ/τ and κ_e_/τ are rising with the increasing carrier concentration. Additionally, the σ/τ and κ_e_/τ for p-type carriers have higher values as compared to n-type. The term thermal power factor is usually referred to the mathematical expression S^2^σ/τ, which characterizes a material’s capability to harvest electrical power from a thermal gradient. The Seebeck coefficient S is in inverse relation, and the electrical conductivity is directly related to carrier concentration. In order to optimize the thermal power factor, careful balancing of these parameters is highly important. In Figure 8d, the PF unveils significant anisotropy when ρ exceeds 6 × 10^19^ cm^-3^. The PF rises meaningfully with the escalating carrier concentration for both n-type and p-type charge carriers, due to enhanced electrical conductivity in both cases.

The capability of a material to convert thermoelectric energy can be assessed by its figure of merit $ZT=\frac{S^{2}\sigma T}{\kappa_{e}+\kappa_{ph}}$ , where κ_ph_ is the lattice thermal conductivity calculated by using the Slack approximation, and T is the temperature in Kelvin. The value of κ_ph_ at 300 K is 8.41 W/mK and decreases to 5.04 W/mK at 500 K. The value of κ_ph_ is much lower than that of the constituent monolayer HfSi_2_N_4_, which is 38.76 W/mK (Das et al., 2025), and Ti_2_CO_2_, which is 21.9 W/mK at 300 K (Gandi et al., 2016). The substantially lower lattice thermal conductivity leads to the higher thermal power factor, making this heterostructure a better thermoelectric material as compared to its constituent monolayers. Figure 8F compares the ZT for p-type and n-type carriers of AD-stacking of Ti_2_CO_2_/HfSi_2_N_4_ as a function of ρ at 300 and 500 K with constant relaxation time approximation (CRTA) at τ = 1 × 10^−13^ s, which has been consistently used in prior theoretical studies for both Ti_2_CO_2_ and HfSi_2_N_4_ monolayers. The CRTA can lead to overestimation of the absolute thermoelectric figure of merit (ZT), a value that and is within the typical range for 2D semiconductors with high carrier mobilities. Despite this limitation, the CRTA remains highly valuable for qualitative screening of thermoelectric candidates, as it reliably captures trends in the Seebeck coefficient which is independent of relaxation time. This is the approach taken in numerous high-throughput DFT studies which assures that CRTA can still provide valuable insight for the comparative studies of the individual monolayers and their heterostructure. Comparing the two temperatures shows that ZT increases with increasing temperature. The larger PF for n-type carrier concentrations at higher concentrations compared to those for p-type results in a consistently higher ZT. The peak value of n-type ZT turns out to be 0.20 (0.82) at a carrier concentration of between 10^20^ to 10^21^ cm^-3^ at 300 K (500 K) temperature.

The primary role of n-type doping in our study is to optimize the thermoelectric performance by increasing the electron concentration, which directly enhances the electrical conductivity and thereby the power factor. However, this would have a significant impact on the OER activity, which is primarily driven by holes (Jang et al., 2025). n-type doping will increase the electron concentration, which can inherently reduce the hole concentration, suppress their mobility, and limit the overall OER efficiency. Furthermore, the n-type doping can also result in the strong adsorption of OER intermediates at the surface, which can also limit the OER performance (Kutlusoy et al., 2022). However, n-type doping can also make it favorable for HER, which requires electrons instead of holes, which can make it a co-catalyst for overall photo/electrocatalytic water splitting depending on its properties after doping. Therefore, there is a fundamental trade-off: the doping condition that optimizes thermoelectric performance (n-type) is not the same as the condition that would optimize photocatalytic OER activity (which would likely benefit from p-type doping).

## Conclusion

We report a detailed theoretical study of four different stacking configurations of vdW-HS Ti_2_CO_2_/HfSi_2_N_4_ having a negligible lattice mismatch of ∼0.23%. Among all the stacking configurations, AD-stacking is the most favorable configuration with the lowest ground state energy and binding energy. The dynamic stability and thermodynamic stability of the AD-stacking configuration have been confirmed by phonon spectra free of imaginary modes across the complete Brillouin Zone and AIMD calculations, respectively. Electronic structure calculations performed using the HSE06 functional with spin-orbit coupling reveal an indirect band gap of 0.89 eV that remains consistent across different stackings. The vdW-HS Ti_2_CO_2_/HfSi_2_N_4_ displays type-I band alignment, which ensures efficient photoexcited electron-hole pair recombination and interlayer exciton formation with electrons transitioning from the VBM to the CBM of Ti_2_CO_2_. From the band edges relative to the vacuum level, the VBM level is in favor of photocatalytic OER and unfavorable for HER, which is also indicated by highly positive and negative values of the ΔG_H_. The vdW-HS Ti_2_CO_2_/HfSi_2_N_4_ demonstrates strong visible absorption and exceptionally high absorption of ultraviolet radiation. The considered vdW-HS outperforms with SLME of ∼32% as compared with other leading thin-film photo-responsive materials. vdW-HS Ti_2_CO_2_/HfSi_2_N_4_ has also displayed outstanding thermoelectric characteristics, which forecast its true potential as a remarkable thermoelectric material. The higher values of S for n-type as compared to p-type carriers suggest that n-type doping is more advantageous for further enhancement of thermoelectric performance. The higher values of PF for n-type carriers at higher concentrations compared to those for p-type result in a consistently higher ZT. These findings position vdW-HS Ti_2_CO_2_/HfSi_2_N_4_ as a promising material for advanced green energy applications.

## Data Availability

The raw data supporting the conclusions of this article will be made available by the authors, without undue reservation.
